# Sex-related differences in vision are heterogeneous

**DOI:** 10.1038/s41598-018-25298-8

**Published:** 2018-05-14

**Authors:** Albulena Shaqiri, Maya Roinishvili, Lukasz Grzeczkowski, Eka Chkonia, Karin Pilz, Christine Mohr, Andreas Brand, Marina Kunchulia, Michael H. Herzog

**Affiliations:** 1 Laboratory of Psychophysics, Brain Mind Institute, EPFL, Lausanne, Switzerland; 2 Laboratory of Vision Physiology, Ivane Beritashvili Center of Experimental Biomedicine, Tbilisi, Georgia; 3 Institute of Cognitive Neurosciences, Free University of Tbilisi, Tbilisi, Georgia; 4 Ludwig-Maximilan University of Munich, Munich, Germany; 5 Department of Psychiatry, Tbilisi State Medical University, Tbilisi, Georgia; 6 School of Psychology, University of Aberdeen, Aberdeen, Scotland UK; 7 Institute for Psychology and Cognition Research, University of Bremen, Bremen, Germany; 8 Institute of Psychology, Faculty of Social and Political Sciences, Bâtiment Geopolis, Quartier Mouline, 1015 Lausanne, Switzerland

## Abstract

Despite well-established sex differences for cognition, audition, and somatosensation, few studies have investigated whether there are also sex differences in visual perception. We report the results of fifteen perceptual measures (such as visual acuity, visual backward masking, contrast detection threshold or motion detection) for a cohort of over 800 participants. On six of the fifteen tests, males significantly outperformed females. On no test did females significantly outperform males. Given this heterogeneity of the sex effects, it is unlikely that the sex differences are due to any single mechanism. A practical consequence of the results is that it is important to control for sex in vision research, and that findings of sex differences for cognitive measures using visually based tasks should confirm that their results cannot be explained by baseline sex differences in visual perception.

## Introduction

In 1894 Ellis^1^ noted that female high school students performed better on verbal memory tasks than male students (for a review, see^2^). In the century since, a large number of studies have confirmed a performance advantage for females on many measures of memory and social cognition^3–5^. Similarly, numerous studies have reported a male advantage on mental rotation and navigation tasks^6–11^ (for meta-analyses, see^12,13^). Sex effects are not limited to cognitive measures. Females outperform males on auditory and somatosensory measures^14,15^.

It is surprising that similar studies in vision research are few and often under-powered^16–19^ (with the notable exception of the well-established male preponderance of red-green color blindness^20,21^ or sex differences in eye movements^22^). For instance, Abramov and colleagues^16^ found that females had lower visual acuity compared to males. This study had only 52 participants, of which 16 were males. Other studies on visual acuity^23,24^, contrast sensitivity^16,18,25^, motion perception^26,27^ and slant estimation^28,29^ had also a limited number of participants and showed mixed results (for a review, see^17^). For example, Brabyn and McGuinness^25^ (n = 39) found that females had higher contrast sensitivity for low spatial frequencies and males had higher contrast sensitivity for high spatial frequencies. Abramov and colleagues^16^ (n = 52) found that males outperformed females in all spatial frequencies, whereas Solberg and Brown^30^ (n = 40) found no sex differences in contrast sensitivity for all spatial frequencies. As Vanston and Strother^17^ point it out, these mixed results might be explained by methodological differences, and as we would like to add, also because of low statistical power. In one large study^31^ (n = 826), visual acuity was measured across the entire age range (5 to 92 years old), and sex differences were found only for children of 5 years old. However, in this study, age was split into ten bins, where such binning reduces the power of the study. In another study, sex-dependent effects were also found in motion perception^26^. However, only young participants from 4 to 24 years old were tested (n = 400) and the results cannot be extrapolated to older participants. Finally, two studies have reported that males outperform females in the Simon task, for both the incongruent (n = 418)^32^ and congruent conditions (n = 176)^33^, respectively. Taken together, these studies reveal mixed and complex effects of sex on visual perception. Moreover, it is clear that a comprehensive study on sex differences is missing from the literature.

Determining if there are sex differences in visual perception is also important because many reports of sex differences in cognition make use of visual tasks. If there are sex differences in vision, these reports of sex differences in cognition could be explained instead by differences in vision, e.g. sex differences in attention might instead be explained by differences in visual filtering^34^.

To address on the need for a comprehensive study of sex differences in visual perception, we analyzed data from more than 870 control participants in several large scale studies of schizophrenia and healthy aging^35–38^ to determine if there are sex differences in any of fifteen common measures of visual perception (such as visual acuity, contrast detection threshold, or motion detection). We found that males outperformed females on six of the fifteen measures, whereas females never outperformed males.

## Methods

### Subjects

We analysed the data of 626 healthy participants recruited from the general population in Tbilisi, Georgia (n = 438), and from Lausanne, Switzerland (n = 188). Most participants had served as control participants in studies on schizophrenia or participated in studies on healthy aging (see^35–38^). All participants had normal or corrected-to-normal visual acuity (≥0.8) as determined by the Freiburg visual acuity test (FrACT; Table 1, Sample A) and were tested, in addition to the FrACT, on vernier duration and visual backward masking. Amongst the 626 subjects, a subset of 200 participants had also been tested in 7 additional tests. Thus, we had performance on 10 tests for these 200 participants (Table 1, Sample B). In addition, we pooled participants from 6 studies testing 5 visual illusions^39^. The set-up and tasks were identical in these studies and the number of participants ranged from 173 to 253 based on the study and illusions tested (all studies did not test the same illusions; see Table 1, Samples C). Participants younger than 18 years old were excluded. Exclusion criteria were drug or alcohol abuse, neurological or other somatic illnesses that could influence the subjects’ mental state. All participants were free from psychiatric axis I disorders.

**Table 1:** Characteristics for the sample of 626 participants (Sample A) that underwent three tests, the subset of 200 participants who performed ten tests (Sample B) and up to 253 participants who were tested with five visual illusions (Samples C; n = 173 to 253).

Samples	Female subjects	Male subjects	p
Sample A (n = 626)	342	284	
Age (mean ± sd)	32.19 ± 15.16	33.09 ± 16.03	p = 0.48
Age range	18–75	18–82	
Sample B (n = 200)	113	87	
Age (mean ± sd)	43.67 ± 23.5	46 ± 25.98	p = 0.51
Age range	18–86	18–90	
Samples C			
Ebbinghaus (n = 209)	93	116	
Age (mean ± sd)	35.37 ± 17.01	33.00 ± 15.25	p = 0.29
Age range	18–73	18–80	
Müller-Lyer (n = 253)	115	138	
Age (mean ± sd)	36.02 ± 16.01	33.47 ± 14.19	p = 0.19
Age range	18–73	18–80	
Ponzo (n = 173)	76	97	
Age (mean ± sd)	28.92 ± 10.18	28.95 ± 9.49	p = 0.99
Age range	18–55	18–54	
Ponzo-Hallway (n = 194)	84	110	
Age (mean ± sd)	36.67 ± 17.44	32.72 ± 14.82	p = 0.10
Age range	18–73	18–80	
Tilt (n = 200)	93	107	
Age (mean ± sd)	38.64 ± 16.28	37.13 ± 14.88	p = 0.50
Age range	18–73	18–80	

The study and methods were carried out in accordance with the guidelines of both universities where participants were tested (in Lausanne, Switzerland and Tbilisi, Georgia). This study was approved by the ethics committee (approval number: 164/14) of the Canton de Vaud in Lausanne, Switzerland and the ethics committee (approval number: 9/07) of the Beritashvili Center of Experimental Biomedicine in Tiblisi, Georgia. All participants signed informed consent forms, were reimbursed for their participation, and were informed that they could quit the experiments at any time.

### Perceptual tests

Extended information and details about the procedure for each test are available in the Supplementary Material.

#### Vernier duration and visual backward masking (n = 626)

**Figure 1:**
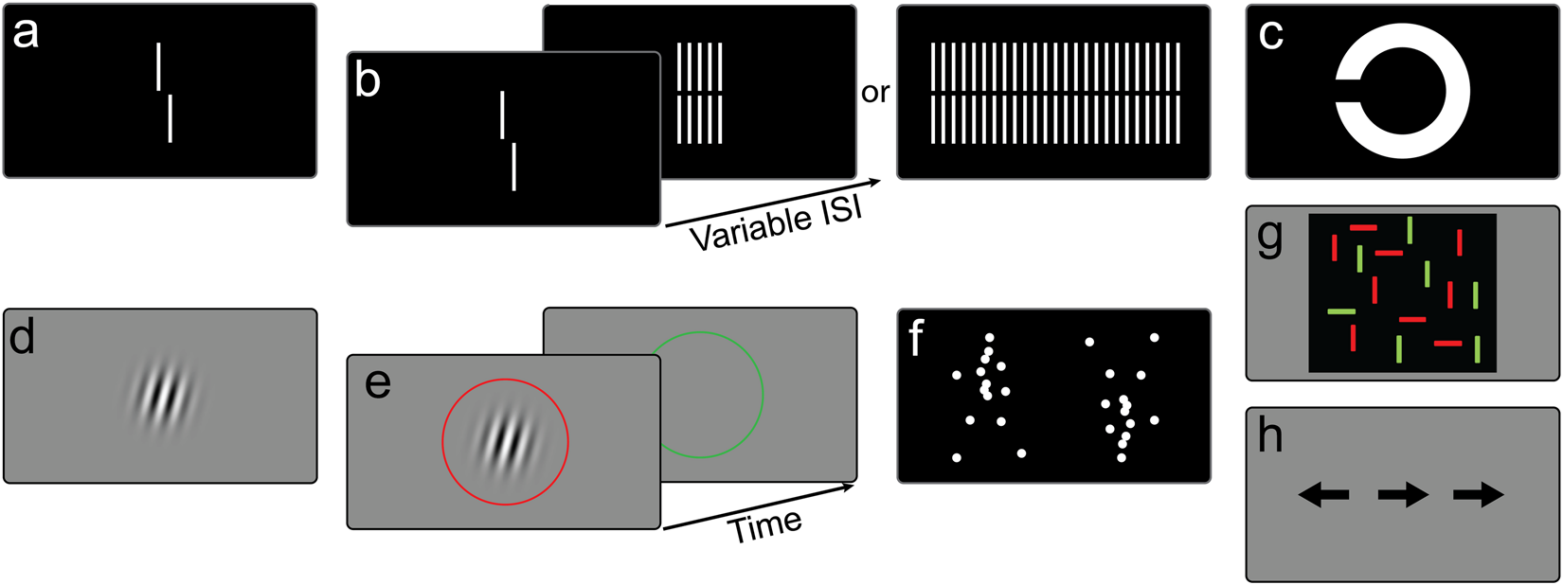
Perceptual tasks: (**a**) vernier duration; (**b**) visual backward masking; (**c**) Freiburg visual acuity test; (**d**) orientation discrimination; (**e**) contrast detection threshold; (**f**) biological motion upright and inverted; (**g**) visual search, (**h**) Simon task.

We followed the same procedure as used previously^40^. We presented verniers composed of two vertical bars that are slightly offset either to the left or right. In a binary task, participants were asked to indicate this offset direction (left/right) by button press. Errors were indicated by an auditory signal. In the masking condition, a grating followed the vernier. The grating comprised either 5 or 25 verniers without offset of the same length and width as the target vernier. Conditions were presented in blocks of 80 trials each (Fig. 1a,b).

In the first step, we presented the vernier without a grating. We aimed to find the shortest vernier duration (VD) for which participants could perform vernier offset discrimination with a threshold below 40” (Fig. 1a; see^40^). We started with vernier durations of 150 ms and reduced durations blockwise until offset discrimination was above 40”.

In the second step, we masked the vernier (for more details, see Supplementary Material A). After the vernier, an inter-stimulus interval (ISI) followed, i.e., a blank screen, and then the grating (Fig. 1b). The grating lasted for 300 ms. We adaptively assessed the target-mask stimulus-onset-asynchrony (SOA = VD + ISI) to yield a performance level of 75% correct responses.

#### Freiburg visual acuity (n = 626)

We followed the same procedure as used previously^41^. Landolt-C optotypes with randomized gap orientations were presented on a computer monitor (Fig. 1c). Participants indicated the direction of the gap (‘up’, ‘down’, ‘left’, “right’) to the experimenter who operated the input device. The size of each optotype changed adaptively following a Best-PEST algorithm.

#### Orientation discrimination (n = 200)

We followed the same procedure as used previously^42^. Participants were asked whether a Gabor patch was oriented clockwise or anticlockwise (Fig. 1d). Auditory feedback was given for incorrect responses. We determined the threshold for which participants achieved 75% correct responses.

#### Contrast detection threshold (n = 200)

We followed the same procedure as used previously^43^. Participants indicated in which of two subsequently presented circles (first red, second green) a Gabor patch was presented (Fig. 1e). A staircase method was used to determine the contrast detection threshold level for which 50% correct responses occurred. Auditory feedback was given for incorrect responses.

#### Coherent motion direction discrimination (n = 200)

We followed the same procedure as used previously^44^. Amongst randomly moving dots, a subset of dots moved together to the left or right (see Figure 6 in Supplementary Material A). Participants discriminated the motion direction and auditory feedback was given. The percentage of target dots, as compared to the amount of distractor dots, varied randomly according to a staircase procedure (PEST). The target starting value was 20% and participants performed 80 trials.

#### Biological motion perception (n = 200)

We followed the same procedure as used previously^45^. Participants were seated 60 cm from the screen and indicated the walking direction of a point-light walker. The walker did not “move” across the screen, but walked on the spot as on a treadmill (Fig. 1f). The walker’s direction of motion was either rightward or leftward. The walker was presented either upright or inverted (conditions were not blocked), for either 200 or 800 ms. Auditory feedback was given for incorrect responses.

#### Simple reaction time (RT) (n = 200)

This task was a modified version of the classic Hick paradigm^46^. Participants were instructed to press a button immediately after a white square (size: 3 arc degrees) had appeared on the screen on a black background. The inter-trial interval (ITI) was varied randomly (minimum 1500 ms, maximum 3500 ms). We determined the RT for the button presses (in ms).

#### Visual Search (n = 200)

We followed the same procedure as used previously^47^. Participants had to search for a green horizontal line segment within an array of red and green lines. Four, 9 or 16 distractor lines were presented in random order. Both speed (in ms) and accuracy (in %) were measured (Fig. 1g).

#### Simon Task (n = 200)

We use a modified version of the visual Simon Task^48^. Participants were subsequently presented with arrows and instructed to respond with the right hand to a right pointing arrow and with the left hand to a left pointing arrow (Fig. 1h). Arrows were presented at three locations on the screen (left, right, or center). In congruent trials, the direction of the arrow matched its location (e.g., left-pointing arrow on the left side of the screen), whereas this was not the case for incongruent trials (e.g., left-pointing arrow on the right side of the screen). To measure the magnitude of the response conflict (Simon effect), we subtracted the RT in the incongruent condition (usually inferior) from the RT in the congruent condition (usually superior). We report these differences in RT in ms.

### Visual illusions

**Figure 2:**
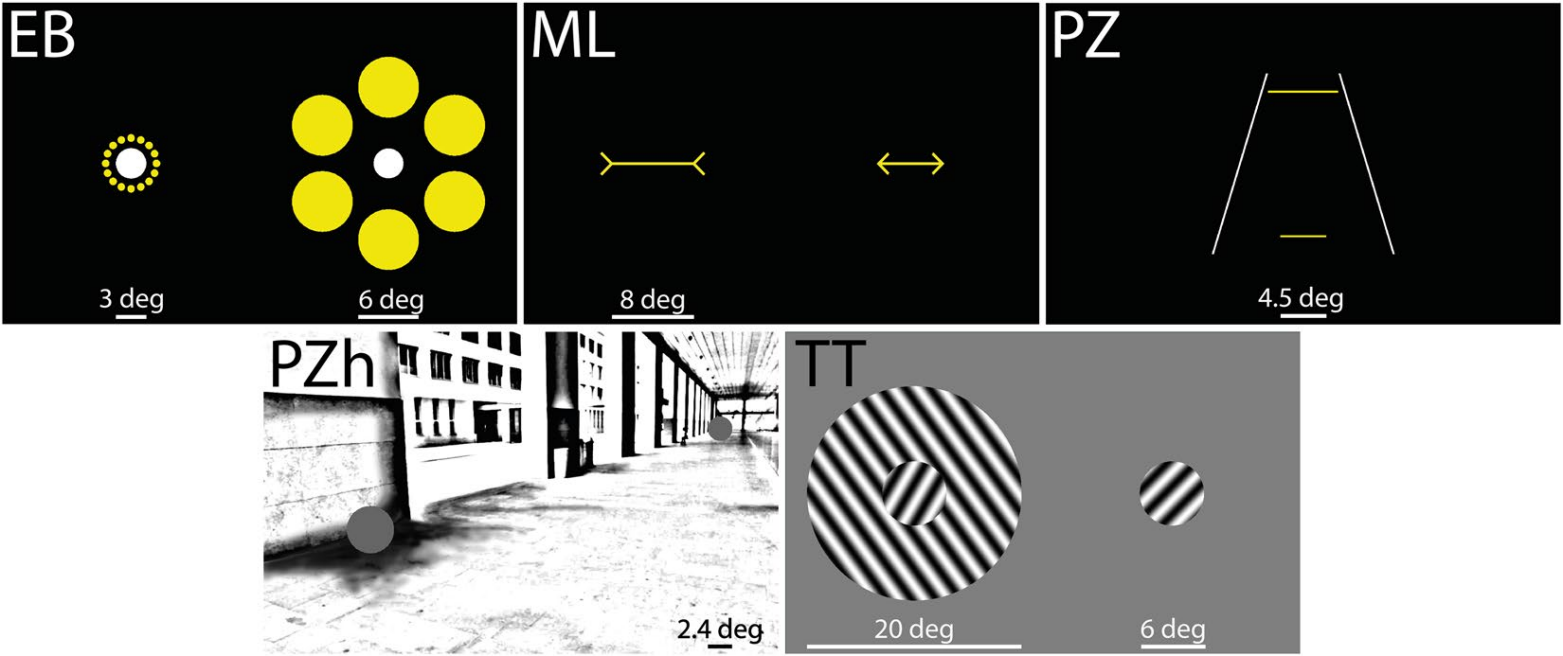
The five illusions tested. In each trial, participants adjusted one element of the stimulus in size, length or orientation to match it with a reference stimulus. The reference item for the Ebbinghaus (EB) was the white left disk and the adjustable element was the right white disk. For Müller-Lyer (ML), the reference item was the left line and the right line was the adjustable element. For Ponzo (PZ), the lower horizontal line was the reference item and the upper one was the adjustable element. For Ponzo-Hallway (PZh), the gray disk in the upper right corner was the reference item and the gray disk in the lower left corner was the adjustable element. In the Tilt illusion (TT), participants adjusted the orientation of the right grating to match the orientation of the inner grating of the stimulus presented on the left side of the image.

We determined illusion magnitudes for the following five visual illusions: the Ebbinghaus (EB), the Müller-Lyer (ML), the Ponzo (PZ), the Ponzo “hallway” (PZh), and the Tilt (TT) illusion. For each illusion, participants adjusted a target to match it in size (EB, PZh), length (ML, PZ) or orientation (TT) to a reference by displacing a computer mouse on its horizontal axis (for more details, see^39^ and Fig. 2).

#### Ebbinghaus illusion (n = 209)

The adjustable disk was surrounded by large inducers of 6 degrees in diameter each. The distance between the center of the adjustable disk and the center of each large inducer was 7.5 degrees. The reference stimulus was a white disk that was 3 degrees in diameter, surrounded by sixteen smaller yellow disks (small inducers), 0.75 degrees of diameter each. The distance between the center of the reference disk and the centers of the small inducers was 2.5 degrees. At the beginning of each trial, the adjustable disk appeared with a random size ranging between 0.0 and 9.2 degrees in diameter. The luminance of the yellow surrounding disks and the white central disk was ≈ 260 cd/m^2^.

#### Müller-Lyer illusion (n = 253)

The adjustable line was always presented with outward-pointing arrows on the right half of the screen and its starting length varied randomly between 0 and 24 degrees. The reference line was 8 degrees long and it was always presented with inward-pointing arrows on the left half of the screen. The lines composing the arrows were 1.5 degrees long. The luminance of lines was ≈ 260 cd/m^2^.

#### Ponzo illusion (n = 173)

We showed two horizontal yellow lines (luminance ≈ 260 cd/m^2^). The adjustable line was the upper one. Its initial length varied randomly from trial to trial, in a range between 0 and 25 degrees of visual angle. The reference line was the lower one and had a length of 4.5 degrees of visual angle. Both lines were centered according to the vertical midline of the screen. In addition, they were shown at 4.75 degrees above (adjustable) and below (reference) the horizontal screen midline. The adjustable and the reference lines were presented together with two white, tilted lines (inducers). The ends of those lines were shown at 5.9 degrees above and below the horizontal screen midline. The distances between the two upper and the two lower line ends were 4.7 and 11.8 degrees respectively (see Fig. 2).

#### Ponzo-hallway illusion (n = 194)

We used a picture of a hallway at the EPFL campus as background image (1920 × 1080 pixel resolution, grayscale). The adjustable disk appeared “at the entrance of the hallway” in the lower-left hand corner at a distance of 16.6 degrees of visual angle from the center of the screen. The reference disk had a diameter of 2.4 degrees. Its center was located “at the end of the hallway” in the top-right hand corner at a distance of 22.2 degrees from the screen’s midpoint. The luminance of both disks was ≈40 cd/m^2^. When participants adjusted the size of the disk, the lowest point of the adjustable disk (its “base”) remained stationary, while its center moved upwards. This size adjustment procedure gave the impression that the disk was anchored at its base with reference to the image background.

#### Tilt illusion (n = 200)

We used two disks that had a diameter of 6 degrees of visual angle. One was the reference disk and the other disk could be adjusted in size. Each disk contained a 0.5 cycles/deg full contrast grating texture. With respect to a vertical orientation, the grating orientation of the reference disk was clockwise, at 33 degrees. This disk was embedded in a larger disk (diameter: 20 degrees of visual angle) containing a grating who’s orientation was tilted 36 degrees counter-clockwise. The grating had the same spatial frequency as the reference disk. The starting orientations of the adjustable disk were randomly spanning from 0 to 360 degrees at each trial.

## Results

### Perceptual tests

Out of the 10 perceptual tests (3 tests for 626 participants and 7 additional tests for 200 participants), males performed significantly better than females in 5 tests: visual acuity, visual backward masking with 25 and 5 gratings, RT, biological motion, and motion direction. The RT test showed a large Cohen’s d effect size (0.7), the other tests showed a small to medium effect size (see Table 2).

**Table 2:** Independent samples t-test for the three perceptual tests in Sample A and seven additional tests in Sample B (see also Table 1).

Test	Participants N	t-test	p	Cohen’s d
Visual acuity (decimals)	626	t(623) = −4.37	<0.001*	0.35
Vernier duration (ms)	626	t(624) = 1.21	0.22	0.09
Visual Back Masking (25) (ms)	626	t(624) = 2.09	0.03*	0.17
Visual Back Masking (5) (ms)	626	t(624) = 2.57	0.01*	0.20
Simple RT (ms)	200	t(198) = 4.97	<0.001*	0.7
Simon test (ms)	200	t(198) = −0.12	0.905	0.01
Contrast (cd/m^2^)	200	t(195) = 0.37	0.71	0.05
Motion Dir (%)	200	t(198) = 2.22	0.03*	0.31
BM Inv 200 (%)	200	t(194) = −1.41	0.16	0.2
BM Inv 800 (%)	200	t(194) = −2.02	0.04*	0.29
BM Up 200 (%)	200	t(194) = −0.76	0.44	0.11
BM Up 800 (%)	200	t(194) = −1.3	0.19	0.18
Orientation (degree)	200	t(198) = 1.72	0.08	0.24
Visual Se. Slope (ms)	200	t(198) = 1.43	0.15	0.2
Visual Se. RT (ms)	200	t(198) = −1.46	0.14	0.21

Out of the three tests, two were significant and out of the additional seven tests, three were significant. Only the RT test showed a large effect size.

**Figure 3:**
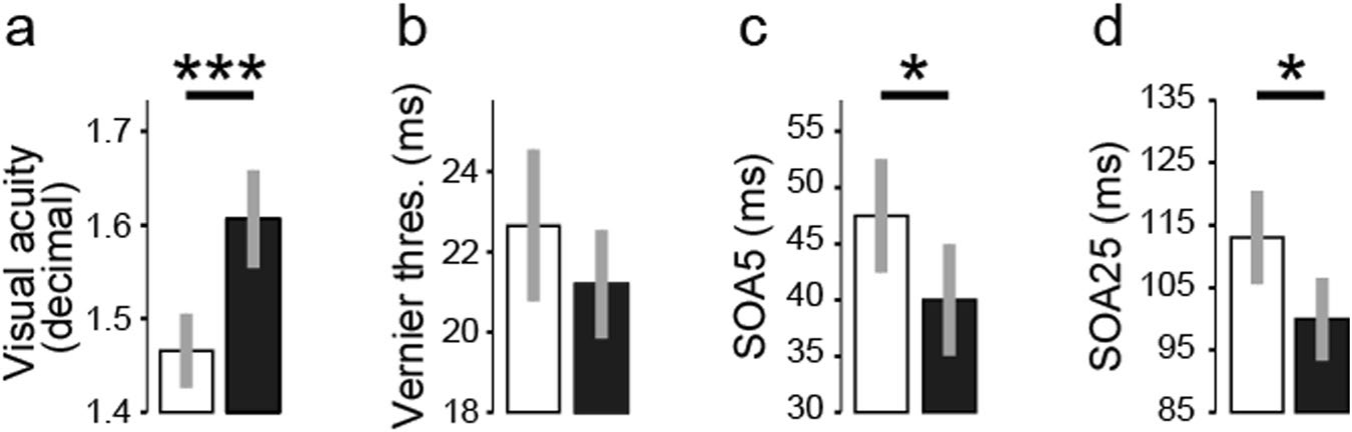
Sample A (Table 1): performance of females (white) and males (black) on the Freiburg visual acuity test (**a**), vernier discrimination (**b**), adding the 25 elements mask (SOA25) (**c**) or the 5 elements mask (SOA5) (**d**). Females have significantly lower visual acuity on The Freiburg visual acuity task compared to males and needed significantly longer SOAs between the vernier and the mask in order to show 75% accuracy rate. The effect size was medium for visual acuity and small for the remaining two significant tests. Error bars represent ± 2 SE of the mean. *p < 0.05, ***p < 0.001.

In detail, we found significant differences in Sample A, with 626 participants, on visual acuity and visual backward masking with both masks, but not for the unmasked vernier (see Fig. 3 and Table 2).

#### Visual acuity

Males had a higher visual acuity compared to females (1.61 vs 1.46; t(623) = −4.37, p < 0.001). The effect size was medium (d = 0.35).

#### Vernier duration

Females (22.66) as compared to males (21.19) did not differ in their vernier duration (t(624) = 1.21, p = 0.22; Table 2).

#### Masking

Using the 25 elements grating, females needed an SOA of 47.78 ms to reach the criterion level of 75% correct answers, whereas males needed an SOA of 39.9 ms (t(624) = 2.09, p = 0.03, d = 0.17) to reach the criterion level (see Table 2). When using the 5 elements grating, both males and females showed longer SOAs than with the 25 elements grating; females again needed longer SOA than males (113.1 vs. 99.93 ms, respectively; t(624) = 2.57, p = 0.01, d = 0.20). To be noted, Cohen’s d was small (0.17) for the SOA25 and SOA5 (0.20), indicating that the low p-value is mainly driven by the large sample size (Fig. 3).

**Figure 4:**
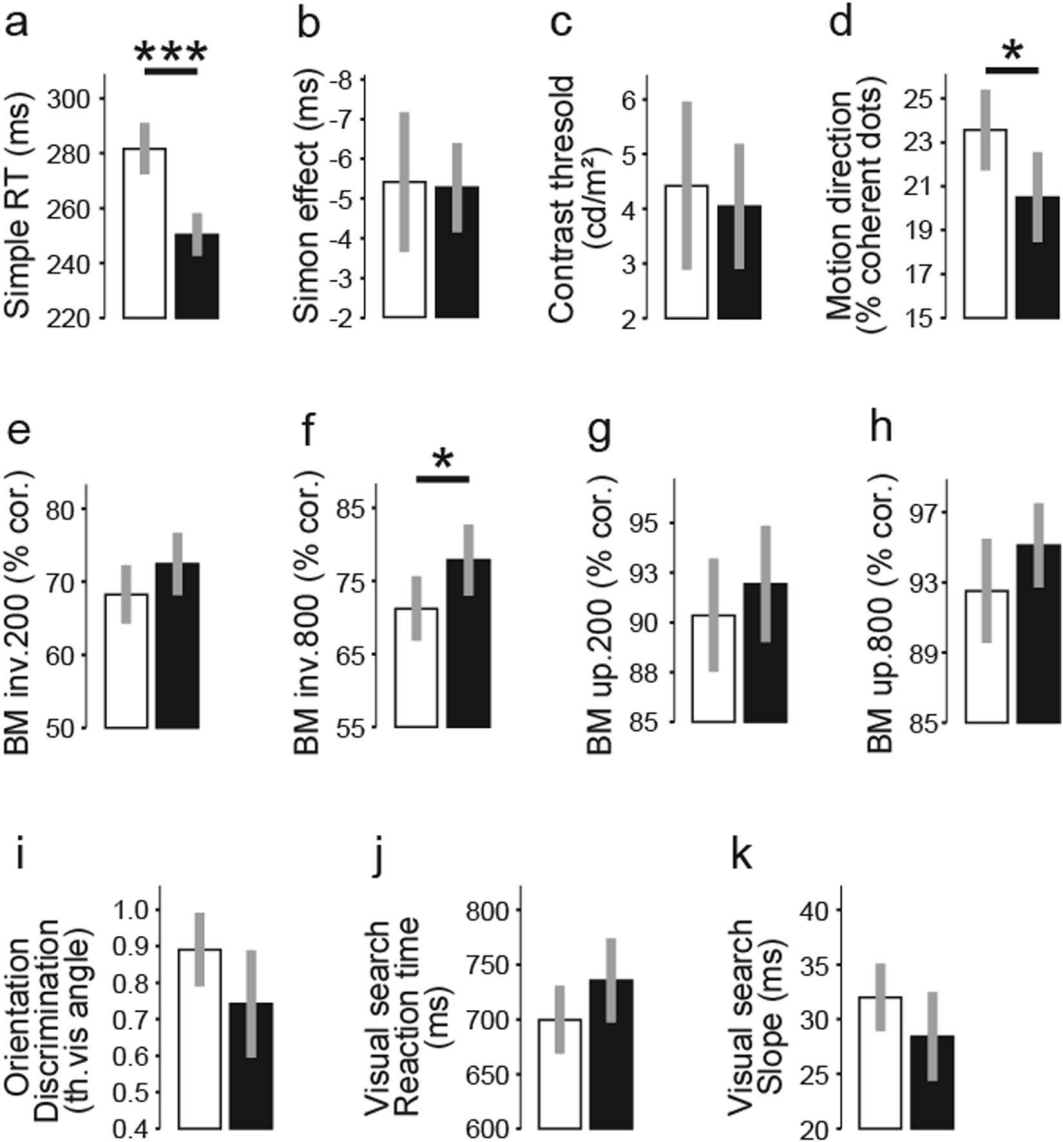
Comparisons for Sample B between females (white) and males (black) for the additional seven tests. We report the results for four conditions for biological motion and two measures for visual search. (**a**) simple RT task, (**b**) Simon effect, (**c**) contrast detection threshold, (**d**) coherent motion detection, inverted biological motion presented for (**e**) 200 ms and (**f**) 800 ms, upright biological motion presented for (**g**) 200 ms and (**h**) 800 ms, (**i**) orientation discrimination, (**j**) RT and (**k**) slope in a visual search task. Females were slower compared to males for the simple RT task and needed a higher number of coherent moving dots in order to perceive motion for the motion direction task. Males outperformed females in perceiving inverted biological motion, but only in the longer (800 ms) presentation condition. Error bars represent ± 2 SE. *p < 0.05, ***p < 0.001.

In Sample B, 200 participants (out of the 626) had performed an additional seven perceptual tests (Table 1). Results from three tests differed between males and females, i.e. RT, biological motion (inverted condition at 800 ms) and motion direction. In all cases, males performed better than females (Fig. 4). We observed a large effect size for the results on the RT test and medium effect sizes for results on biological motion and motion direction (Table 2, Fig. 4).

### Visual Illusions

**Figure 5:**
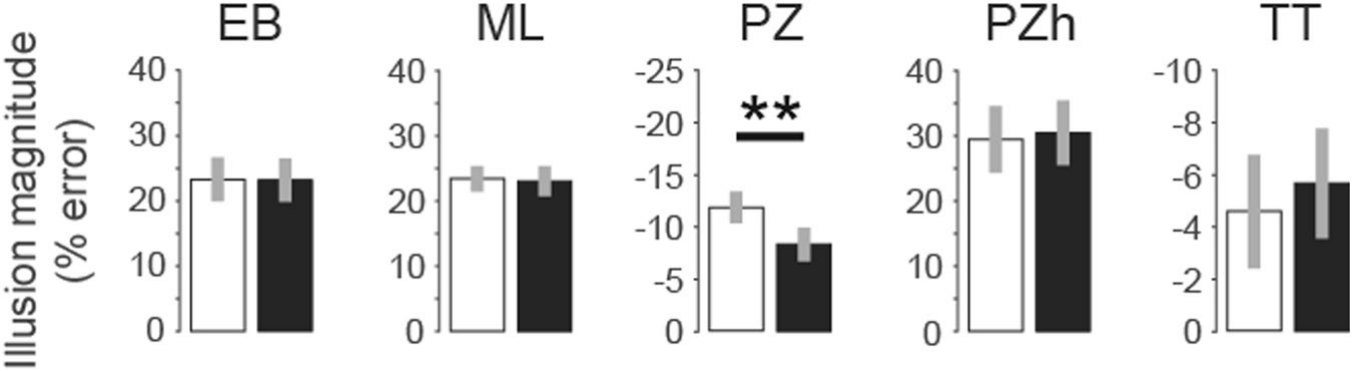
Illusion magnitudes as percentage of error for females (white) and males (black). Illusion magnitudes were calculated as the mean difference between the adjustable target and the reference element. This difference was divided by the reference value and multiplied by 100. For the Tilt illusion, the mean difference between the target item and the reference element was divided by the orientation difference between the surround and the center, i.e, 69 degrees. Thus, one degree corresponds to 1.45% of error. Error bars represent ± 2SE, *p < 0.05, ***p < 0.001.

For Sample C (Table 1), we found a significant sex difference for the Ponzo illusion with a medium effect size (Table 3, Fig. 5; t(170) = −3.15, p = 0.002, d = 0.24). Females were 3.5% more susceptible to the illusion than males (−11.8 vs −8.3%). No significant sex difference was found for the remaining four visual illusions (Table 3).

**Table 3:** Sample C (see also Table 1).

Test	Participants N	t-test	p	Cohen’s d
Ebbinghaus	209	t(203) = 0.04	0.96	0.01
Müller-Lyer	253	t(249) = 0.24	0.81	0.02
Ponzo	173	t(170) = −3.15	0.002*	0.24
Ponzo-Hallway	194	t(186) = −0.29	0.77	0.27
Tilt	200	t(187) = 1.27	0.21	0.11

Independent t-test results for the five visual illusions. Cohen’s d was calculated using the pooled standard deviation. We found that females were more susceptible to the Ponzo illusion than males.

## Discussion

Sex differences in audition and somatosensation have been well established (e.g., females outperform males in tactile acuity^15^, temperature detection^15^, and baroreflex frequency^14,15^) but, similar data for vision are sorely lacking. We report here the first large scale examination of sex differences in visual perception. Using fifteen different visual tasks and more than 870 participants, we found that males significantly outperformed females in simple RT, visual acuity, visual backward masking, motion direction detection, biological motion, and the Ponzo illusion. We did not find significant sex differences for contrast detection threshold, visual search, orientation discrimination, the Simon effect, and four of five visual illusions. For the paradigms with significant sex differences, effect sizes ranged from small to medium (0.17 to 0.35), except for the RT task, where the effect size was large (0.7).

We found no obvious pattern linking the paradigms with significant sex differences. For example, tasks with significant sex differences included both elementary (motion direction, RT, visual acuity, visual backward masking) and complex (biological motion and illusions) visual processing. Even between similar tasks, the results were heterogeneous. For example, we found a significant sex difference for the Ponzo illusion but not for the other four illusions, even though both the Ebbinghaus and Ponzo illusions are spatial in nature. Likewise, we found a clear sex difference in visual acuity (d = 0.35) and visual backward masking (d = 0.25), but no sex differences in contrast detection (d = 0.05) or vernier duration (d = 0.09), despite all of these tasks being low-level in nature. Moreover, we obtained markedly different sex effects for the vernier duration test (p = 0.22) and the visual backward masking test (p = 0.03) even though both measures involve vernier offset discrimination. Even more surprisingly, biological motion discrimination with an inverted walker and a duration of 800 ms showed a significant sex difference (d = 0.29), but the other three biological motion tasks did not (d = 0.11 to 0.2). All four biological motion tasks used the same observers, suggesting that sex differences may exist in vision, but they are rather idiosyncratic. It seems unlikely that a general effect of hormonal status (estrogen and/or testosterone level)^49^ or brain size differences^50–52^ could easily account for this complex pattern of findings.

Our results are in line with studies demonstrating no correlations between similar paradigms in visual perception^22,39,53–55^. For example, upright and inverted biological motion are not correlated in older participants^55^ and out of 5 visual illusions, only the Ponzo and the Ebbinghaus illusion are significantly correlated^39^. Finally, Goodbourn and colleagues^56^ found no correlation between magnocellular tasks (for a review, see^54^).

Our inability to find more sex differences is unlikely to be due to poor power. We computed a sensitivity power analysis (using GPower^57^ with α = 0.05, β = 0.80, 2-tailed), and for our sample size of 626 participants, the minimal detectable effect size was Cohen’s d = 0.22, which is usually considered small. We did not correct for multiple testing so as to not obscure existing sex differences. For this reason, it may even be that some of our significant results are false positives. However, the important finding of our study is not the specific sex differences we found, but rather the heterogeneity of our results.

As mentioned, sex differences can depend on age^26,31^ so to discount age as a potential confound, we included it as a covariate in a set of analyses (see Supplementary Material B). While there were strong age effects for most tasks, the effect of age did not account for any sex differences. For example, even when our analyses were restricted to younger participants, females had lower visual acuity than males. Similar results were found for biological motion.

It is important to emphasize that visual tasks also rely on non-visual processes. It is therefore possible that some of the differences we report may be non-visual in nature. For example, the sex differences we observed in reaction time may reflect, to some extent, differences in motor processing (in many motor paradigms, such as finger tapping, males are faster)^58–60^. Furthermore, the Simon task also depends on selective attention^32^. However, we did not find significant sex differences in the Simon task, indicating that both the visual and attentional aspects of this task do not differ between males and females.

Our results stand in contrast to many previous studies of sex differences in visual perception. For example, Ishigaki and Miyao^31^ found sex differences in visual acuity only in children 5 years of age. There were no significant differences in the other nine age groups. Closer examination of their results seems to suggest an overall advantage for male participants, but their choice to analyze each age group separately reduced the power of their analyses and may have hindered their ability to detect a significant effect. Schrauf and colleagues^26^ observed significant sex differences in motion perception whereas we did not, but these results cannot easily be compared, as their study included participants from 4 to 24 years old and our participants were 18 to 90 years old. Stoet^61^ found that males were better than females in visual search whereas we did not. However, our visual search task entailed search for a horizontal green line among red and green distractor lines, whereas Stoet^61^ used an orange T shape among blue T-shaped distractors. In addition, the number of distractors was different in the two studies. Finally, we did not find any sex differences in the Simon task, contrary to Stoet and colleagues^32^ and Evans and Hamspon^33^, who found that males were better than females. In our paradigm, the arrows were presented on the left, the right or in center of the screen, whereas in the studies of Stoet^32^ and Evans and Hamspson^33^, the arrows were presented to the left or right only. Furthermore, performance was determined separately for the congruent and incongruent conditions in these studies, whereas we determined performance in the Simon task by subtracting RT on the incongruent trials from RT on the congruent trails. To better compare these results, we analyzed performance separately for incongruent trials and congruent trials, and did not find any differences between males and females on either the congruent trials (t(197) = 1.15, p = 0.25) or the incongruent trials (t(197) = 1.51, p = 0.13). It is unclear why our results differ from previous studies, but it is possible that the small methodological differences we describe may have a large effect, and further studies should explore these effects in more detail.

Our results have methodological, mechanistic, and conceptual implications. Methodologically, even though the sex differences we found are not large, between subjects designs should control for the ratio of female:male participants, or include sex as a factor in statistical analyses. In particular, tasks involving speeded responses should test for sex differences, as random assignment can lead to substantially variable sex ratios in studies with relatively small samples, and our results suggest that this could have a large effect. Mechanistically, as mentioned, there was no obvious pattern linking the paradigms with significant sex differences, implying no single or simple cause. For this reason, specific mechanistic explanations about sex differences in visual paradigms, such as hormonal status, need to be interpreted with care. Finally, the conceptual implications of our study are paramount. In general, sensation precedes perception, which precedes cognition, and differences at early stages of this chain may propagate to later stages such that sex differences in cognition could reflect differences in vision. For example, a sex difference in visual filtering might manifest in attention or cognitive load^34^.

In summary, we used, for the first time, a battery of 15 tests to investigate sex differences in vision with a large sample of participants. We found that, for about a third of these tests, females performed significantly worse than males. In no paradigm did females outperform males. However, our effect sizes were rather small overall, and the tasks with significant differences were heterogeneous. Even small methodological differences, such as stimulus rotation, can abolish sex differences. Therefore, small effect sizes, low power, and varying methodology may explain why results in the literature are often mixed. The heterogeneity of significant results makes it unlikely that there is one common cause explaining sex differences in visual perception. Research using visual tasks should control for sex in their cohorts, should understand that sex differences have a complex, multi-factorial basis, and should consider visual mechanisms first before concluding a “higher order” locus of sex differences in cognitive tasks.

## Electronic supplementary material

Supplementary Material
