# Supplementary material for: Sex-related differences in vision are heterogeneous
Source: Sci Rep. 2018 May 14;8:7521. doi: 10.1038/s41598-018-25298-8 (PMC5951855; doi:10.1038/s41598-018-25298-8)
Supplement: Supplementary file 1 — Supplementary Material [file 41598_2018_25298_MOESM1_ESM.docx]

Sex-related differences in vision are heterogeneous

Albulena Shaqiri^1a*^, Maya Roinishvili^2,3a^, Lukasz Grzeczkowski^4^, Eka Chkonia^3,5^, Karin Pilz^6^, Christine Mohr^8^, Andreas Brand^7^, Marina Kunchulia^2,3^, Michael H. Herzog^1^

^1^ Laboratory of Psychophysics, Brain Mind Institute, EPFL, Lausanne, Switzerland

^2^ Vision Research Laboratory, Beritashvili Centre of Experimental Biomedicine, Tbilisi, Georgia

^3^ Institute of Cognitive Neurosciences, Agricultural University of Georgia, Tbilisi, Georgia

^4^ Ludwig-Maximilan University of Munich, Germany

^5^ Department of Psychiatry, Tbilisi State Medical University, Tbilisi, Georgia

^6^ School of Psychology, University of Aberdeen, Scotland, UK

^7^ Institute for Psychology and Cognition Research, University of Bremen, Bremen, Germany

^8^ Institute of Psychology, Faculty of Social and Political Sciences, Bâtiment Geopolis, Quartier Mouline, 1015 Lausanne, Switzerland

a: both authors contributed equally

* Corresponding author: Albulena Shaqiri, Laboratory of Psychophysics, Brain Mind Institute, School of Life Sciences, Ecole Polytechnique Fédérale de Lausanne, Station 19, CH-1015, Lausanne, Switzerland; tel: (+41) 21-693-1741, fax: (+41) 21-69-31749, e-mail: albulena.shaqiri@epfl.ch

SUPPLEMENTARY MATERIAL

# Supplementary Material A

## Detailed tasks descriptions

### Vernier task and backward masking

This task has been frequently used in our laboratory, and measures cortical rather than retinal processing^1,2^. Stimuli are presented from a distance of 5 meters in a dimly illuminated room. The stimuli are white (100 cd/m^2^) on a black background (see also Figure 1). In a first step, we present vernier stimuli consisting of two vertical bars of 10` (arc min) of length which are offset in the horizontal direction. Participants indicate via button press the offset direction of the lower bar compared to the upper bar (left or right). The offset direction is chosen randomly. Errors are indicated by an auditory signal. Participants do 80 trials. For each observer, we determine the individual vernier duration (VD, in ms) to reach 75% correct responses using a staircase procedure (for details, see^1^).


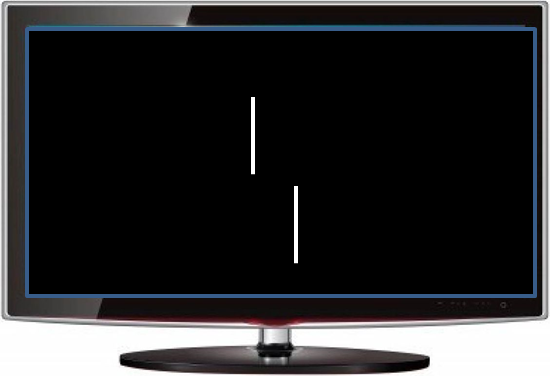


Figure 1. Example of a typical vernier stimulus, used to determine individual vernier duration.

Once the individual vernier duration is established (20 ms is the minimal duration), the vernier is followed in the second step by a variable inter-stimulus interval (ISI), i.e. a blank screen, and then a grating for 300 ms.. We vary the ISI adaptively using a staircase procedure^3^. The grating consists of either 5 or 25 elements without offset of the same length as the target vernier (see also Figure 2). The horizontal distance between grating elements was about 3.33′. The outcome measure is the stimulus onset asynchrony (SOA = Individual vernier duration + ISI, in ms). The starting value of the SOA was 200 ms and then it either increased or decreased in order to find the individual threshold for each participant. Participants performed 80 trials.


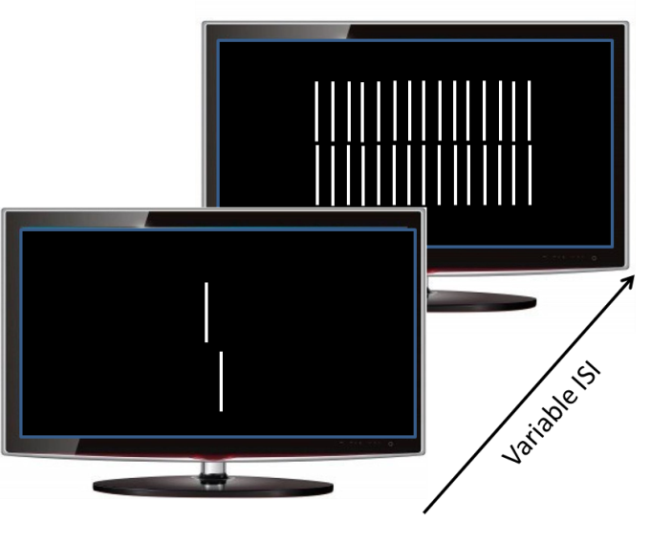

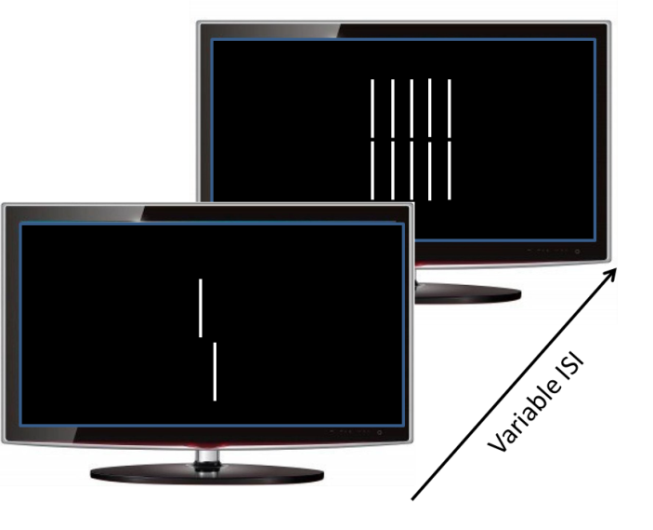


a)

b)

Figure 2. Example of a typical backward masking paradigm, where a vernier is followed by a 5 (a) or a 25 line grating (b).

The five element grating (= SOA5) leads to stronger masking than the 25 element grating (= SOA25) even though the five element grating is contained in the 25 element grating^4^. This difference in masking strength indicates that a substantial part of the masking power is not of retinal origin because retinal processing is mainly determined by the sheer amount of light, e.g., the number of grating elements presented.

### Freiburg Visual Acuity Test (= FrACT)

This test has been developed and described in detail by Bach^5^, and has been validated in various studies^6,7^. FrACT is a computerized visual acuity (=VA) test capable of presenting very large Landolt-C optotypes with randomized gap orientations on a computer monitor. Participants sit 5 meters from the computer screen. In this study, the white optotypes are presented on a black computer screen and participants have to indicate the direction of the opening of the optotype (Figure 3). There are four possible answers (‘up’, ‘down’, ‘left’, ‘right’), and participants are instructed to verbalize their responses, while the experimenter operates the input device. The size of each optotype presented always targets the currently most probable VA threshold, calculated on the basis of all previous responses following a Best-PEST algorithm^8^.


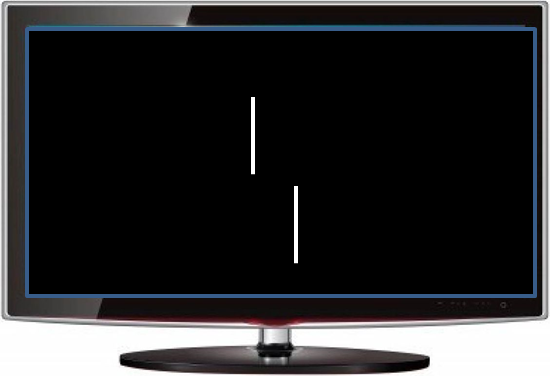


Figure 3: Example of a typical Freiburg visual acuity test display (FrACT), with a white optotype being opened to the right.

### Contrast detection threshold

This task measures contrast sensitivity based on a similar procedure and stimuli used in Lahav^9^. We used what is described as “*The transient method*” in Lahav and colleagues^9^. A Gabor with spatial frequencies ranging from 1.5 to 6 cycles per degree is presented in the middle of the screen. Stimuli are presented from a distance of 2 meters in a dimly illuminated room. The task is a two forced choice task. After a fixation cross, the screen is blank (grey) for 500 ms then a red circle of 2 arc degrees appears (for 320 ms) in the middle of the screen. The screen is blank again for 800 ms then a green circle appears (for 320 ms). Only one of the two circles has a Gabor patch at its center (see Figure 4) and the circle which will contain the Gabor is chosen randomly. Participants have to indicate in which of two subsequently presented circles they could detect the Gabor patch by pressing the correspondingly colored button. A staircase method was used to determine the contrast threshold level (in candela per m^2^, or cpm^2^) at 50% correct. Auditory feedback was given in case of incorrect answers. Participants performed 80 trials.


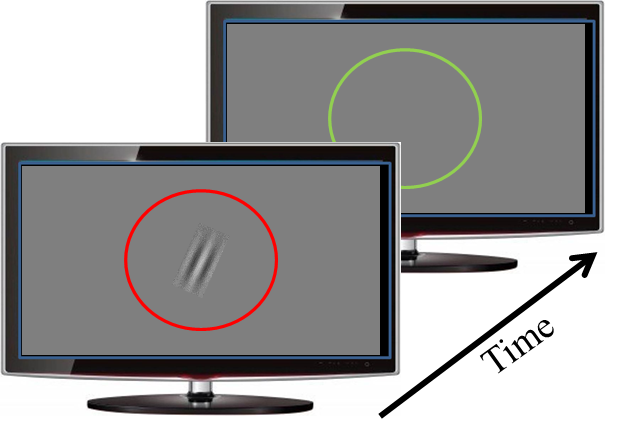


Figure 4. Example of a typical contrast detection task display, with the Gabor being presented in the red circle.

### Orientation discrimination

This is an adapted version of the orientation discrimination task as used by Tibber and colleagues^10^. Participants are seated 2 meters from the computer screen, and required to decide whether the target stimulus (a Gabor patch determined by Michelson contrast of 34%, average luminance of 21 cd/m2, spatial frequency of 4 cyc/deg) is oriented clockwise or anticlockwise (see *Figure 5*). Participants gave their response by pushing a right (for right-oriented Gabors) or a left (for left-oriented Gabors) button. Participants performed 80 trials. Auditory feedback is given when an incorrect response is registered. The outcome measure is the perceptual threshold in degrees of visual angle.


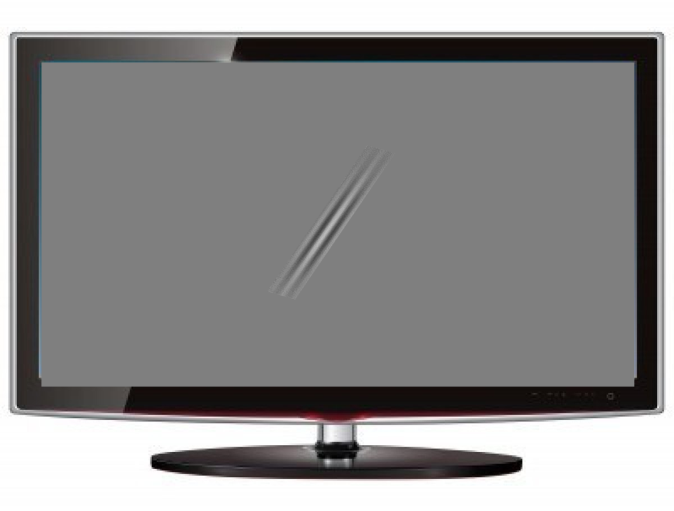


*Figure 5.* Example of a typical orientation discrimination task display, with the Gabor tilted to the right.

### Motion direction

This task measures global motion perception, and the stimuli and procedure are based on a paper by Roudaia and colleagues^11^. Participants are seated 2 m in front of the screen, and have to judge the general motion of dot patterns. Stimuli are white dots presented on a black background. On each trial, the pattern displays dots that are either displaced uniformly to the right or to the left (= targets), or move independently from each other (= distractors). The percentage of target dots as compared to the amount of distractor dots varies randomly according to a staircase procedure (= PEST). The target starting value is 20%. Auditory feedback is given in case of incorrect responses. Participants perform 80 trials.


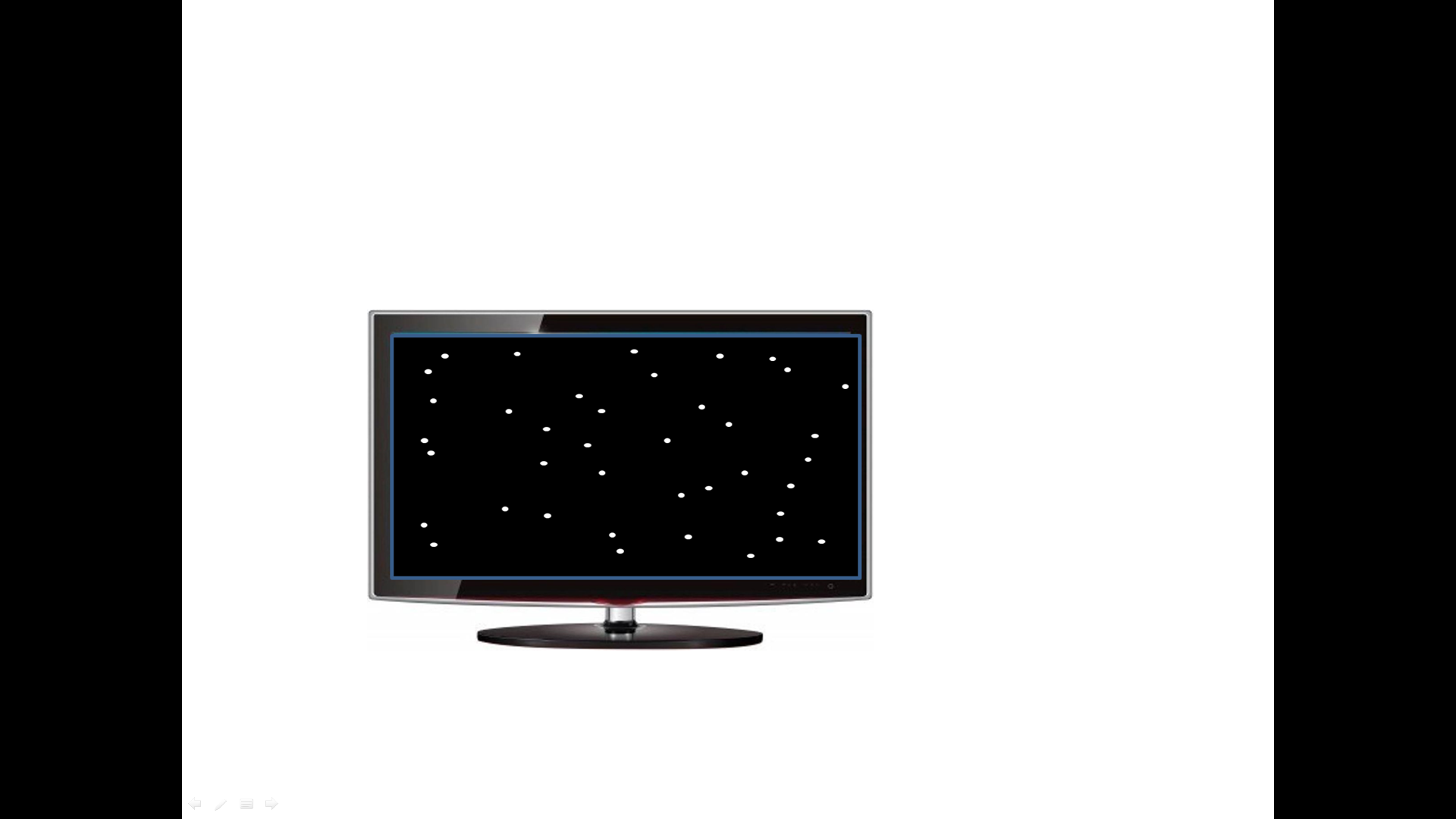


Figure 6. Example of the motion direction task. Participants need to decide whether the white moving dots are moving to the left or right while ignoring the distractors.

### Biological motion perception

This task measures biological motion perception, and is based on a paper by Pilz and colleagues^12^. Participants are seated 60 cm from the screen, and have to indicate the walking direction of a point-light walker by button press (left or right, see also Figure 7). The walker does not move across the screen, but rather appears to walk in place as if on a treadmill. The walker’s direction of motion is either rightward or leftward, and the walker is either presented upright or inverted (conditions are not intermixed), for either 200 or 800 ms. The accuracy rates (in %) give an indication of biological motion perception performance. Participants perform 80 trials. The dots forming the walkers are white and presented on a black background.


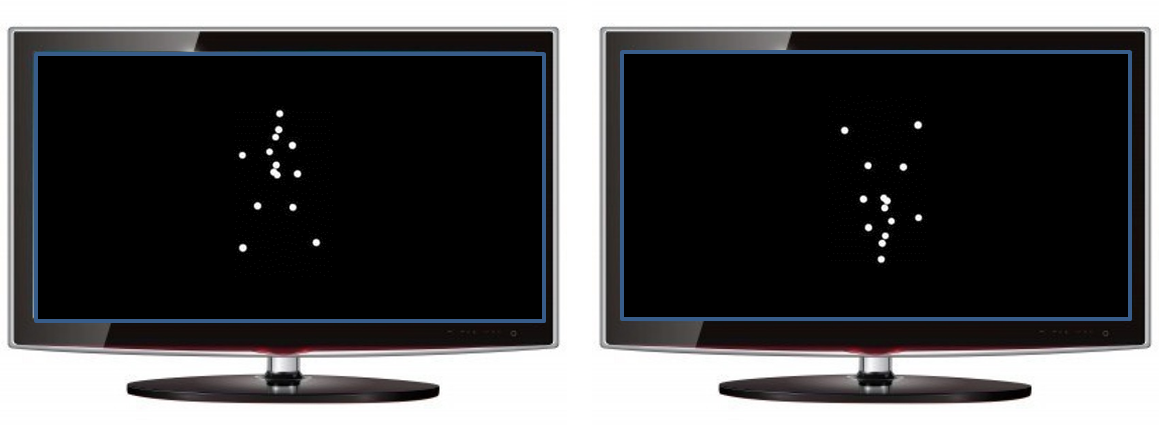


b)

a)

Figure 7. Example of a typical biological motion task display, with an upright walker walking to the left (a), and an inverted walker walking to the right (b).

### Simple RT

This task was a modified version of the classical Hick-paradigm^13^. Participants are seated at 2 meters distance to the computer screen and are instructed to press a button immediately after a white square (3 arc degrees size) appears on the screen on a black background (see Figure 8). The inter-trial interval is varied randomly to prevent participants from predicting when a square would appear, but with a minimal ISI of 1500 ms. RT (in ms) is used as a measure of behavioral response time/speed of information processing. Participants performed 80 trials.


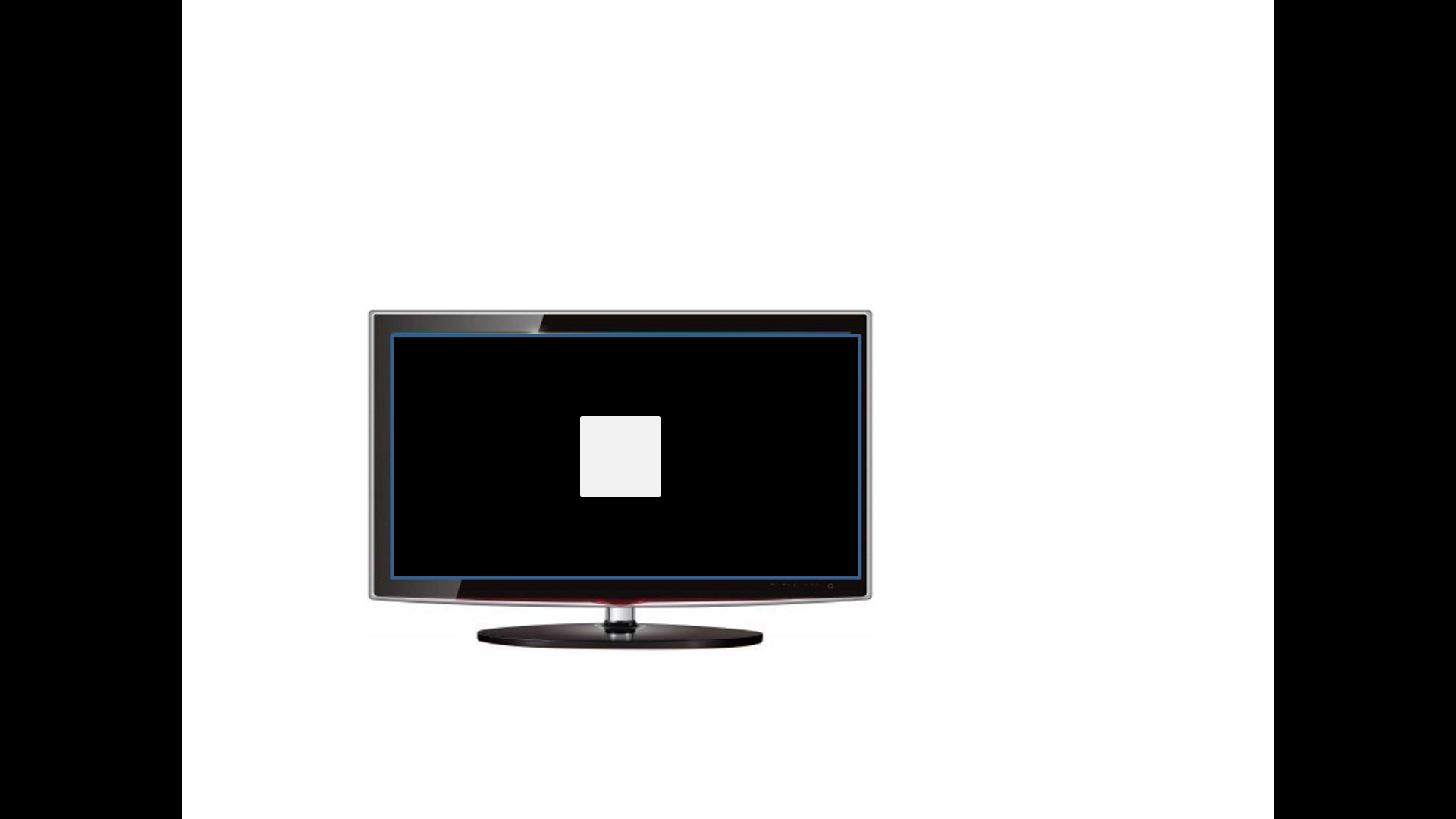


Figure 8. Example of a simple RT trial. Participants responded to the appearance of a white square as soon as possible by button press.

### Visual Search Task

***
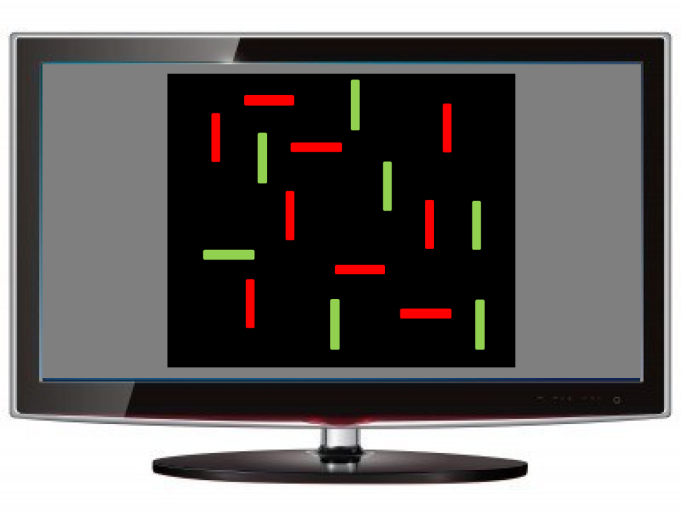
***In this task, participants have to search for a green horizontal line segment within an array of distractors (see also Figure 9). The display elements are presented on a uniform black background and grey surrounding. They consist of green or red line segments. The stimulus field consists of 4, 9 or16 line segments in random order. In order to detect the target, a conjunction of features (color and orientation) is required. Both speed (in ms) and accuracy (in %) in finding the target are measured.

*Figure 9.* Example of a typical visual search task display (set size 16), with the target being present.

### Simon Task

In our study, we use a modified version of a visual Simon-task found to be sensitive to aging effects^14^. Subjects are seated 2 meters observation distance to the computer screen, and are subsequently presented with black arrows on a grey background and instructed to respond with the right hand to a right pointing arrow; respond with left hand to a left pointing arrow. Arrows are presented on three locations on the screen (left, right or center). There are two trial types. In congruent trials the direction of the arrow matches its location (e.g. left-pointing arrow on the left side of the screen), whereas this is not the case for incongruent trials (e.g. left-pointing arrow on the right side of the screen). To measure the magnitude of the response conflict (= Simon effect) for % correct responses an index is calculated by subtracting performance in the incongruent condition (usually inferior) from performance in the congruent condition (usually superior), and vice versa for RT´s. This way, positive values also indicate a normal response conflict effect, whereas negative values indicate an unusual response conflict effect. Auditory feedback is given in case of incorrect responses. Participants perform 80 trials.


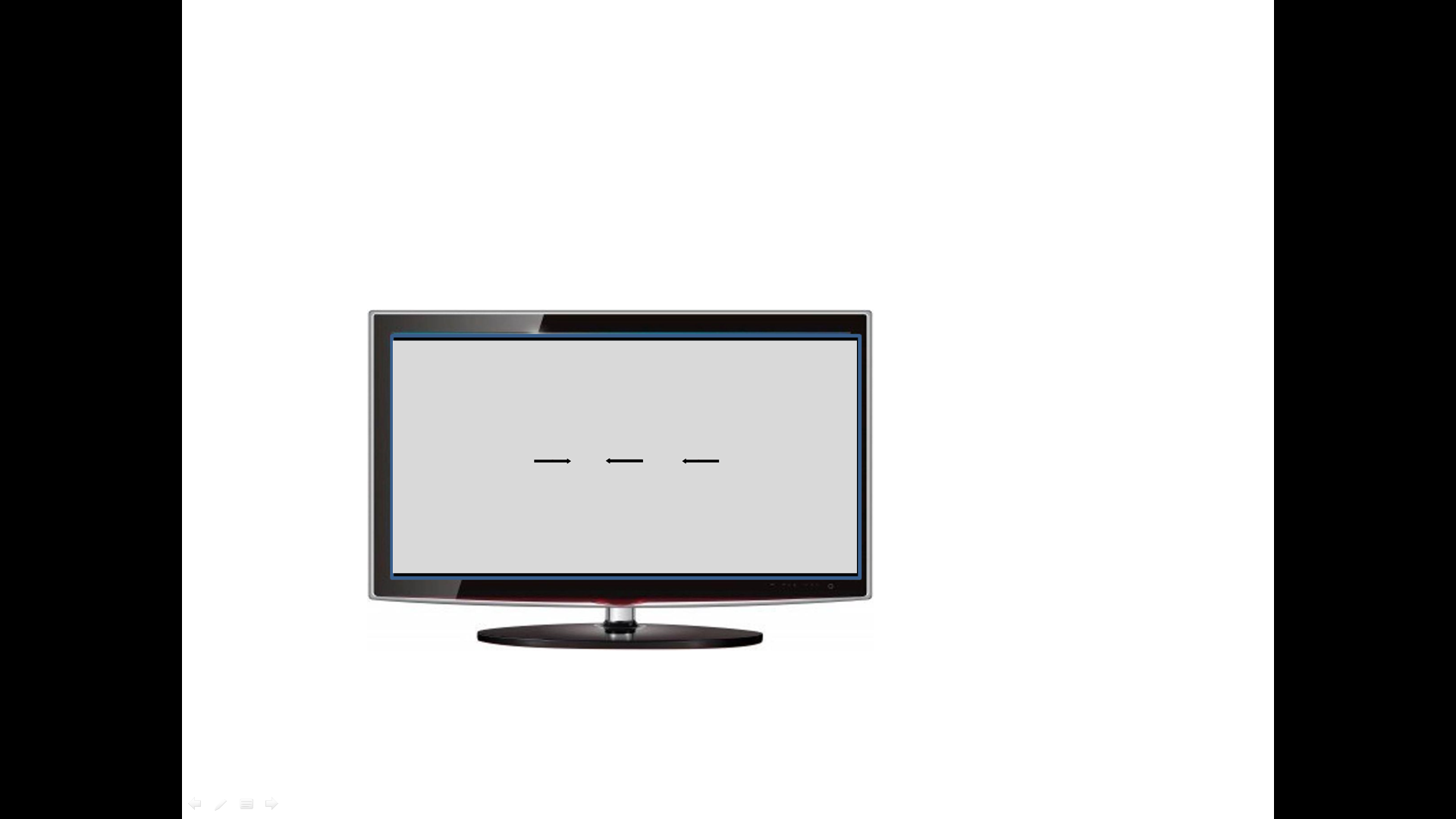


Figure 10. Example of a Simon task trial. Participants have to respond if the arrow is pointing to the left or right.

### References

1. Herzog, M. H., Kopmann, S. & Brand, A. Intact figure-ground segmentation in schizophrenia. *Psychiatry Res.* **129,** 55–63 (2004).

2. Roinishvili, M., Chkonia, E., Stroux, A., Brand, A. & Herzog, M. H. Combining vernier acuity and visual backward masking as a sensitive test for visual temporal deficits in aging research. *Vision Res.* **51,** 417–423 (2011).

3. Taylor, M. M. & Creelman, C. D. PEST: Efficient Estimates on Probability Functions. *J. Acoust. Soc. Am.* **41,** 782–787 (1967).

4. Herzog, M. H. & Koch, C. Seeing properties of an invisible object: feature inheritance and shine-through. *Proc. Natl. Acad. Sci. U. S. A.* **98,** 4271–5 (2001).

5. Bach, M. The Freiburg Visual Acuity Test-Automatic Measurement of Vis... : Optometry and Vision Science. *Optom. Vis. Sci.* **73,** 49–53 (1996).

6. Schulze-Bonsel, K., Feltgen, N., Burau, H., Hansen, L. & Bach, M. Visual Acuities ‘Hand Motion’ and ‘Counting Fingers’ Can Be Quantified with the Freiburg Visual Acuity Test. *Investig. Opthalmology Vis. Sci.* **47,** 1236 (2006).

7. Wesemann, W. Visual acuity measured via the Freiburg visual acuity test (FVT), Bailey Lovie chart and Landolt Ring chart. *Klin. Monbl. Augenheilkd.* **219,** 660–7 (2002).

8. Treutwein, B. Adaptive psychophysical procedures. *Vision Res.* **35,** 2503–2522 (1995).

9. Lahav, K., Levkovitch-Verbin, H., Belkin, M., Glovinsky, Y. & Polat, U. Reduced Mesopic and Photopic Foveal Contrast Sensitivity in Glaucoma. *Arch. Ophthalmol.* **129,** 16 (2011).

10. Tibber, M. S., Guedes, A. & Shepherd, A. J. Orientation Discrimination and Contrast Detection Thresholds in Migraine for Cardinal and Oblique Angles. *Investig. Opthalmology Vis. Sci.* **47,** 5599 (2006).

11. Roudaia, E., Bennett, P. J., Sekuler, A. B. & Pilz, K. S. Spatiotemporal properties of apparent motion perception and aging. *J. Vis.* **10,** 5–5 (2010).

12. Pilz, K. ., Bennet, P. J. & Sekuler, A. . Effects of aging on biological motion discrimination. *Vision Res.* **50,** 211–219 (2010).

13. Hick, W. E. On the rate of gain of information. *Q. J. Exp. Psychol.* **4,** 11–26 (1952).

14. Castel, A. D., Balota, D. A., Hutchison, K. A., Logan, J. M. & Yap, M. J. Spatial attention and response control in healthy younger and older adults and individuals with Alzheimer’s disease: Evidence for disproportionate selection impairments in the simon task. *Neuropsychology* **21,** 170–182 (2007).

**Supplementary Material B**

#### Perceptual tests and age

When using age as a covariate, we found that visual acuity is variable with age: older participants have a lower visual acuity compared to younger participants. As Figure 1A shows, the sex differences are only present at early ages; from age 40 and on, there are no sex differences in visual acuity. The age-sex interaction is significant (F(1, 621)=5.18, p=0.02), but it is mainly driven by the younger participants.


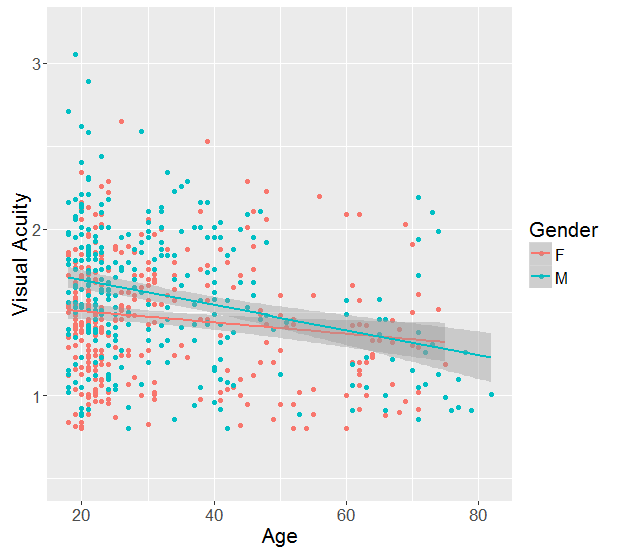

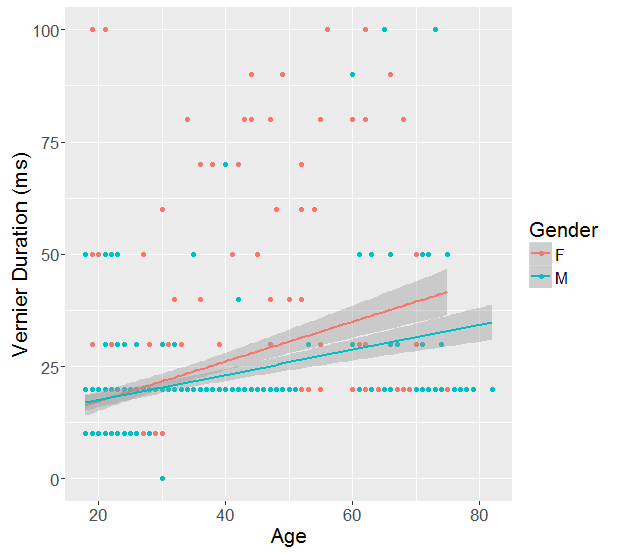


A

B


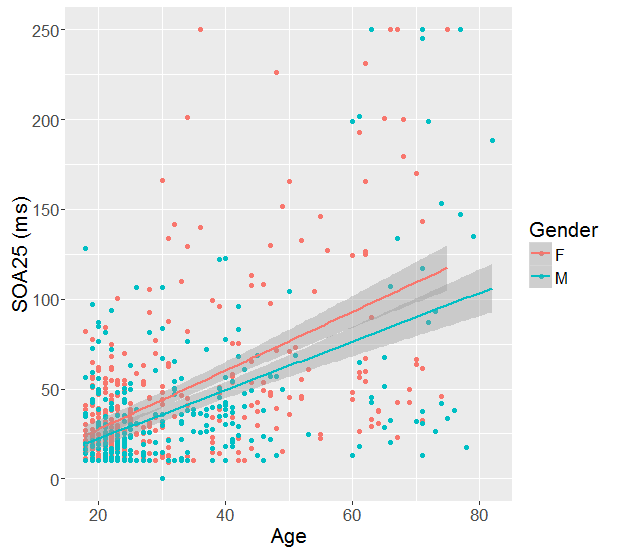

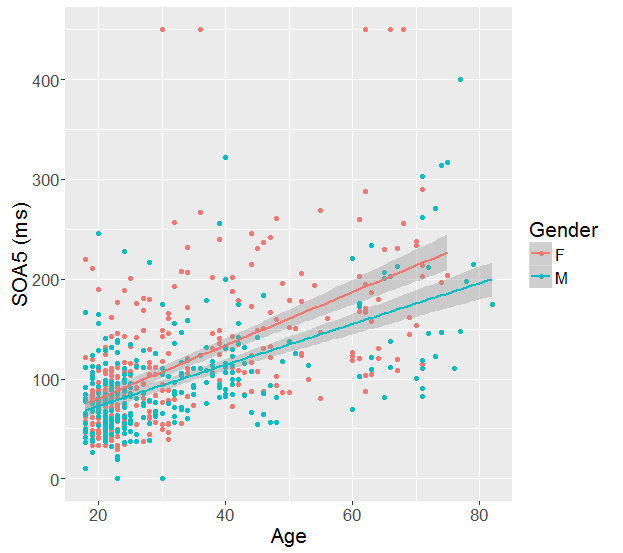


C

Figure 1: Perceptual changes with age. **A**. For visual acuity, the sex differences are mainly due to younger participants. Those differences disappear from age 40 and on. **B**. Vernier duration changes based on age. From age 50 and onwards, females have a longer vernier duration compared to males. **C.** Participants’ performance on SOA25 and SOA5 based on age. For SOA5 (right), there is sex difference (such as females perform worse compared to males) that appears after the age of 30 years old. The grey area represents 95% of confidence level.

The interaction between age and sex was significant for VD (F(1, 622)=5.27, p=0.02), driven by the performance of older participants. Figure 1B shows that from the age of 50, sex differences become significant and females have longer VD compared to males.

For visual backward masking (Figure 1C), older participants have a longer SOA25 compared to younger (F(1,624 )=230.09, p<0.001), but there is no age-sex interaction (F(1,622)=2.09, p=0.14). For SOA5, there was a main effect of age (F(1, 624)=308.01, p<0.001) but also an interaction age-sex (F(1, 622)=5.34, p=0.02). Figure 3C shows that females perform worse on the task with increasing age.

We also included age as a covariate for the 7 perceptual tests and found that for all tests, age had a statistically significant effect (with all p values <0.025). Indeed, performance in all tests deteriorated with age. Nevertheless, when comparing sex and age for the RT task, we found that younger males and females were not different, it is only with age that the sex difference increased, with females being slower than males (F(1, 197)=5.7, p=0.025). A similar pattern was also true for motion direction perception (F(1,97)=20.07, p<0.001). For the biological motion inverted test at 800 ms, it was the opposite effect: young males were statistically better than young females, but this sex difference disappeared with increasing age, such that overall, older participants performed worse than younger participants, but both sexs performed alike with older age (60 and onwards).
